# Boosting care and knowledge about hereditary cancer: European Reference Network on Genetic Tumour Risk Syndromes

**DOI:** 10.1007/s10689-018-0110-6

**Published:** 2018-10-09

**Authors:** Janet R. Vos, Lisette Giepmans, Claas Röhl, Nicoline Geverink, Nicoline Hoogerbrugge, ERN GENTURIS, Nicoline Hoogerbrugge, Marjolijn Ligtenberg, Marleen Kets, Rolf Sijmons, Gareth Evans, Emma Woodward, Marc Tischkowitz, Eamonn Maher, Verena Steinke-Lange, Elke Holinski-Feder, Thierry Frebourg, Claude Houdayer, Rosalie E. Ferner, Jan Lubinski, Karolina Ertmanska, Svetlana Bajalica Lagercrantz, Emma Tham, Ignacio Blanco Guillermo, Gabriel Capella, Joan Brunet Vidal, Conxi Lázaro, Judith Balmaña, Vincent Bours, Eric Legius, Pierre Wolkenstein, Bela Melegh, Carla Oliveira, Manuel Teixeira, Bruce Poppe, Kathleen Claes, Hector Salvador Hernandez, Stefan Aretz, Isabel Spier, Rianne Oostenbrink, Mateja Krajc, Ana Blatnik, Evelin Schröck, Sirkku Peltonen, Marja Hietala, Chrystelle Colas

**Affiliations:** 1 Department of Human Genetics, Radboud University Medical Center, P.O. box 9101, 6500 HB Nijmegen, The Netherlands; 2 Research B.V, University Medical Center Groningen, University of Groningen, Groningen, The Netherlands; 3 Patient representative, European Patient Advocacy Group, European Reference Network on Genetic Tumour Risk Syndromes (ERN GENTURIS), Vienna, Austria; 4 Department of Urology, Radboud University Medical Center, Nijmegen, The Netherlands; 5 Coordinator European Reference Network on Genetic Tumour Risk Syndromes (ERN GENTURIS), Nijmegen, The Netherlands

**Keywords:** Hereditary cancer, Cross border health care, European Reference Network, Rare diseases, Syndrome, Genetic

## Abstract

Approximately 27–36 million patients in Europe have one of the ~ 5.000–8.000 known rare diseases. These patients often do not receive the care they need or they have a substantial delay from diagnosis to treatment. In March 2017, twenty-four European Reference Networks (ERNs) were launched with the aim to improve the care for these patients through cross border healthcare, in a way that the medical knowledge and expertise travels across the borders, rather than the patients. It is expected that through the ERNs, European patients with a rare disease get access to expert care more often and more quickly, and that research and guideline development will be accelerated resulting in improved diagnostics and therapies. The ERN on Genetic Tumour Risk Syndromes (ERN GENTURIS) aims to improve the identification, genetic diagnostics, prevention of cancer, and treatment of European patients with a genetic predisposition for cancer. The ERN GENTURIS focuses on syndromes such as hereditary breast cancer, hereditary colorectal cancer and polyposis, neurofibromatosis and more rare syndromes e.g. *PTEN* Hamartoma Tumour Syndrome, Li Fraumeni Syndrome and hereditary diffuse gastric cancer.

## The pan-European approach to rare diseases

### The current state of rare diseases in Europe

Health systems in the European Union (EU) aim to provide high-quality, cost-effective care. For rare and complex diseases, this is a challenge due to the fragmented knowledge about these diseases [1, 2]. Hence, patients with a rare disease often do not receive the care they need or experience significant delay to the correct diagnosis and treatment. Across Europe there is substantial variation in health care for patients with a rare disease, especially considering prognosis, quality of life, healthcare costs, available guidelines and patient information. A rare disease is defined as a condition that affects fewer than 1 in 2,000 people. It is estimated that 6–8% of all people someday in their lives will face a rare disease [1]. In the EU there are approximately 27–36 million people who experience daily suffering from one of the 5000 to 8000 known rare diseases.

The ERNs are focused on providing a system of care, in which the medical knowledge and expertise travels across the borders, rather than the patients. In case it is unavoidable the patient will travel to the nearest foreign expertise centre. In addition to care, the ERNs provide a platform for education, training, patient empowerment, the development of guidelines and research activities.

### The objective of European Reference Networks

European reference networks (ERNs) are virtual networks of medical experts from all over Europe. ERNs are focused on rare or complex diseases that require highly specialized treatments and a combined effort of expert knowledge and resources. The ERNs aim to improve the care of patients with a rare disease [3, 4]. The European Commission Expert Group on Rare Diseases, in collaboration with EURORDIS, a European alliance of rare disease patient organisations, has identified themes in which all rare diseases will be classified. In March 2017, 24 ERNs were appointed to these themes by the Board of Member States of the European Union, which oversees the ERNs [3, 5]. These 24 ERNs unite more than 900 expert teams from more than 300 hospitals and 150 patient representatives from 25 European member states in the area of one or more themes [4–6]. At national level, many expertise centres for rare disease have been recognized by their government in the last few years. The procedures and criteria for the establishment of an ERN and the selection of members are regulated in EU legislation. To become a member of an ERN, a hospital or healthcare institute needs national endorsement and must meet a number of general requirements set by the European Commission (such as recognition by the national government) and a number of theme-specific criteria for each ERN (such as minimum number of patients and demonstrable facts related to expertise).

### Information and access to European Reference Networks

In the years to follow a healthcare professional in the EU can use the expertise of the relevant ERN via one of the (national) expertise centres involved in the ERN of interest [7]. Patients can request advice from the network through their local medical specialist. In practice, this means that the healthcare professional refers the patient to the nearest expertise centre in their own country, and then this expertise centre will, if necessary, present the case to other experts in the ERN.

The patients will be asked informed consent by their referring healthcare professional for discussing their case via the Clinical Patient Management System: the secure web-based application functioning as the platform where healthcare professionals from the European Reference Networks (ERNs) discuss real patient cases. The informed consent form enables patients to participate and profit from the ERN at 3 levels: (1) to have their medical data shared with healthcare professionals in the ERN so that they may work together to support the patient’s care; (2) to have their de-identified data being included in one or more ERN database or registry; (3) to be contacted about the possibility to participate in a specific research project.

## The ERN for genetic tumour risk syndromes (ERN GENTURIS)

### The state of affairs in the field of hereditary cancer in Europe

Hereditary tumour syndromes are often characterized by an increased risk for common tumours such as breast and colorectal cancer, by the occurrence of cancer at a younger age and by tumours at multiple locations. More than forty different forms of hereditary cancer syndromes are known [8]. Currently in Europe the majority of patients with a genetic tumour risk syndrome is not yet recognized as such [9–13]. However, if the hereditary predisposition is known in time, risk management options can be provided to prevent cancer from occurring or to detect cancer at an early stage. Often this is related to a better prognosis in both the patient and his/her family as also other family member could have inherited the genetic predisposition causing the syndrome. In addition, patients with hereditary cancer can benefit from a treatment adapted to the hereditary predisposition [14–18].

### The role of ERN GENTURIS in the field of hereditary cancer

The European Reference Network on Genetic Tumour Risk Syndromes (ERN GENTURIS) is the ERN in the field of hereditary cancer. ERN GENTURIS aims at all patients in Europe with a known hereditary cancer predisposition by integrated, multidisciplinary activities in the fields of care, guidelines, education and research directed towards improved identification, genetic diagnostics, cancer prevention and treatment [19].

ERN GENTURIS is coordinated by N. Hoogerbrugge, MD, PhD from the Radboud university medical center and currently brings together 23 centres in the field of hereditary cancer from 12 European member states, as well as seven patient representatives [19]. Within ERN GENTURIS, the rare or complex syndromes are divided into four themes: (1) Neurofibromatosis; (2) Hereditary colorectal cancer and Polyposis; (3) Hereditary breast and ovarian cancer; (4) Other rare, mainly malignant, hereditary tumour risk syndromes such as *PTEN* Hamartoma tumour Syndrome (PHTS), Li-Fraumeni syndrome, hereditary melanoma (FAMM), and hereditary stomach cancer.

The governmental structure of ERN GENTURIS includes a National Coordinators Board consisting of national representatives for each of the participating member states. The national coordinators act as contact with all HCPs, national patient and professional organizations and networks in their country. They will disseminate centrally established output and if necessary “translate” this into national products. For member states where no HCP is found that fulfil the GENTURIS criteria, an affiliated partner will act as national representative for ERN GENTURIS. In this way, all member states will be involved in ERN GENTURIS and will have access to guidelines and registries. The ERN will collaborate with other European stakeholders like current scientific collaborations, medical societies and patient representatives.

More information about ERN GENTURIS and the participating expertise centres and partners and their specific expertise can be found on the website of ERN GENTURIS (http://www.genturis.eu).

### Network activities

The network is organized via closed biannual network meetings and monthly executive committee meetings and task force meetings. Task forces are formulated to organize and stimulate six key functions of the ERN: (1) Coordination and management, (2) Organization of care, (3) Guidelines, outcomes and quality, (4) Education and training, (5) Research, data registration and grants, and (6) Patient empowerment, communication and dissemination.

*Organization of care* The ERN GENTURIS is an expert reference network for patients with or suspected of having a genetic tumour predisposition syndrome. Only a physician can consult the network and/or refer a patient with any question related to the health care pathway of these syndromes. This includes topics as identification, diagnostics, treatment and risk management. A triage system is in place to manage these incoming requests by complexity, and facilitate a suitable action from an answer from an individual local or international ERN expert to an answer after multidisciplinary team discussion among experts within the ERN GENTURIS.

*Guidelines* The ERN GENTURIS will use her expertise to develop guidelines for care. The approach used to develop guidelines will be based on the NICE process, including the GRADE, AGREE, and Delphi methodology. For each syndrome a committee will be selected, consisting of a core group and an extended group. The core group will consist of three people who cover the defined disciplines of the core multidisciplinary team of the syndrome. The extended group will cover relevant disciplines of the extended multidisciplinary team, including a patient representative. Each guideline will relate to one of six modules of the clinical pathways: (1) General description of the syndrome, (2) Identification of patients, (3) Surveillance and follow-up, (4) Treatment of symptoms, (5) Prophylactic treatment, and (6) Psychosocial counselling.

*Registry* The ERN GENTURIS registry covers a standardised minimal dataset on genotype and phenotype which will be captured following international classifications and ontology’s. Patients who were referred to the ERN GENTURIS and who gave permission on their ERN informed consent form for data registration will be included in the registry. Rules of governance are being established that will include procedures for reviewing data requests, data access and data sharing of pseudonymised data.

## Conclusion

In the near future medical specialists can call upon the ERNs with healthcare questions related to patients with rare or complex diseases. European collaboration is essential, both for the care that is currently needed as for the continuous and sustainable improvement of care. In this respect, the ERNs will provide an important stimulus to improve the life of many patients with rare diseases.
